# Effects of sensory distraction and salience priming on emotion identification in autism: an fMRI study

**DOI:** 10.1186/s11689-021-09391-0

**Published:** 2021-09-23

**Authors:** Genevieve Patterson, Kaitlin K. Cummings, Jiwon Jung, Nana J. Okada, Nim Tottenham, Susan Y. Bookheimer, Mirella Dapretto, Shulamite A. Green

**Affiliations:** 1 Department of Psychology, University of Denver, Denver, USA; 2 Jane and Terry Semel Institute of Neuroscience and Human Behavior, Department of Psychiatry and Biobehavioral Sciences, University of California Los Angeles, Los Angeles, USA; 3 Department of Psychology, University of North Carolina at Chapel Hill, Chapel Hill, USA; 4 Havard Medical School, Boston, USA; 5 Department of Psychology, Columbia University, New York, USA; 6 Ahmanson-Lovelace Brain Mapping Center, 660 Charles E. Young Drive South, Los Angeles, CA 90095 USA

**Keywords:** Autism, Emotion, fMRI, Sensory processing, Sensory over-responsivity

## Abstract

**Background:**

Social interaction often occurs in noisy environments with many extraneous sensory stimuli. This is especially relevant for youth with autism spectrum disorders (ASD) who commonly experience sensory over-responsivity (SOR) in addition to social challenges. However, the relationship between SOR and social difficulties is still poorly understood and thus rarely addressed in interventions. This study investigated the effect of auditory sensory distracters on neural processing of emotion identification in youth with ASD and the effects of increasing attention to social cues by priming participants with their own emotional faces.

**Methods:**

While undergoing functional magnetic resonance imaging (fMRI), 30 youth with ASD and 24 typically developing (TD) age-matched controls (ages 8–17 years) identified faces as happy or angry with and without simultaneously hearing aversive environmental noises. Halfway through the task, participants also viewed videos of their own emotional faces. The relationship between parent-rated auditory SOR and brain responses during the task was also examined.

**Results:**

Despite showing comparable behavioral performance on the task, ASD and TD youth demonstrated distinct patterns of neural activity. Compared to TD, ASD youth showed greater increases in amygdala, insula, and primary sensory regions when identifying emotions with noises compared to no sounds. After viewing videos of their own emotion faces, ASD youth showed greater increases in medial prefrontal cortex activation compared to TD youth. Within ASD youth, lower SOR was associated with reduced increased activity in subcortical regions after the prime and greater increased activity in the ventromedial prefrontal cortex after the prime, particularly in trials with noises.

**Conclusions:**

The results suggest that the sensory environment plays an important role in how ASD youth process social information. Additionally, we demonstrated that increasing attention to relevant social cues helps ASD youth engage frontal regions involved in higher-order social cognition, a mechanism that could be targeted in interventions. Importantly, the effect of the intervention may depend on individual differences in SOR, supporting the importance of pre-screening youth for sensory challenges prior to social interventions.

**Supplementary Information:**

The online version contains supplementary material available at 10.1186/s11689-021-09391-0.

## Background

Autism spectrum disorders (ASD) are characterized by difficulties with social interaction, which relies heavily on the ability to process emotional expressions. Individuals with ASD consistently show altered face processing [1, 2]; yet, why this occurs is still not well understood. One area that is understudied is the effect of sensory distraction on face processing. Sensory over-responsivity (SOR), defined as an extreme sensitivity to sensory stimuli such as touch or sound, is very common in ASD, occurring at rates of 56–78% [3–8]. SOR is also associated with greater social difficulties [9, 10]. Given the essential role that face processing plays in social communication, understanding the impact of sensory distraction upon emotional face processing is highly relevant for developing more effective interventions for ASD that take into account real-world social settings in which there is substantial competing sensory information.

Neuroimaging research can provide insight into the mechanisms underlying these processes, which can then inform intervention. Although behavioral studies of emotional face processing in ASD have generated mixed results, fMRI and EEG studies have consistently revealed atypical neural activation during emotional face processing in individuals with ASD. However, the direction of the effects and the regions affected have varied depending on both the context of the task and participant characteristics (for reviews, see [1, 11, 12]). For example, multiple studies have reported lower activation in the fusiform face area during face processing tasks in individuals with ASD [13–19], but in studies where participants were explicitly instructed to look at a fixation cross in the center of the presented faces or were directed to look at the eyes, participants with ASD displayed typical fusiform activity [20, 21]. Differences have also been found between implicit and explicit facial emotion recognition tasks, with participants with ASD displaying lower fusiform activity during implicit but not explicit facial emotion recognition [22]. In addition, activity in the fusiform gyrus and amygdala during face processing has been reported to correlate with time spent fixating on the eyes for individuals with ASD, increasing to similar levels as control subjects in the case of the fusiform and increasing to higher levels than control subjects in the case of the amygdala [13]. Therefore, the brain mechanisms underlying face processing in ASD may depend greatly on where attention is directed, which in turn may depend on the extent to which individuals are distracted by extraneous sensory stimuli.

SOR has generally been associated with greater social challenges within individuals with ASD at a behavioral, physiological, and neural level. Multiple studies have shown associations between parent-reported SOR and poorer social functioning in children with ASD on measures including the social awareness subscale of the Social Responsiveness Scale (SRS), the socialization subscale of the Vineland Adaptive Behavior Scales (VABS), and the DSM-IV social scales [9]. In children with ASD, higher SOR was related to higher cortisol levels during a peer-interaction paradigm, indicating higher levels of physiological stress during social interaction [23]. At a neural level, previous work from our lab demonstrated that youth with ASD showed reduced activity in auditory language areas and mentalizing frontal regions when completing a social task (interpreting communicative intent) in the presence of simultaneous mildly aversive tactile stimuli, whereas typically developing youth demonstrated increased activity in these same regions [24].

To date, very few studies have looked specifically at the relationship between face processing and sensory processing atypicalities. One recent study found that atypical event-related potential (ERP) correlates of face processing in siblings of children with ASD were associated with greater parent-reported social communication and sensory difficulties [25]. Another study found that self-reported sensation avoiding was correlated with greater right fusiform activity while looking at faces in adults with ASD but not in controls [26]. However, to our knowledge, the neural basis of facial emotion identification in ASD in the presence of competing aversive sensory stimuli has not yet been examined.

One possible explanation for the relationship between sensory and social challenges in ASD is an over-attribution of salience to extraneous sensory information at the expense of social cues. This is supported by findings that SOR in youth with ASD is associated with reduced neural habituation to mildly aversive sensory stimuli in primary sensory cortices as well as in regions related to salience and affective valence such as the amygdala [27–29]. Sustained responses to sensory information in the amygdala suggest sustained attention and salience attribution. Furthermore, SOR severity in youth with ASD is related to atypical resting-state connectivity in the salience network, an intrinsic brain network thought to be involved in selecting which of many competing external and internal stimuli an individual attends to [30, 31]. Specifically, youth with higher SOR showed increased resting-state functional connectivity between salience network hubs and primary sensorimotor regions but decreased connectivity between salience network and visual association areas involved in social cognition [32]. According to this framework, interventions could reduce the impact of sensory distractions on social functioning either by reducing the salience of the sensory information or increasing the salience of the social information.

There has been some evidence showing that directing attention to relevant cues in social tasks can improve behavioral performance and activate normative or compensatory neural circuitry. For example, Li and Tottenham [33] showed that neurotypical adults with higher autism traits were initially slower to identify emotional expressions. However, this difference was no longer observed when participants were primed with videos of their own faces making emotional expressions. In addition, our prior work showed that directing attention to key social cues led to children with ASD engaging medial prefrontal cortex (mPFC) and maintaining activation in social processing regions during a social inference task [34] even with sensory distraction [24]. Increased activation in mPFC was associated with lower levels of social difficulties [34] and sensory over-responsivity [24]. Taken together, these studies suggest that increasing the salience of as well as attention to social cues can improve social functioning even in the context of extraneous sensory distracters, likely through engagement of the prefrontal cortex.

The role of mPFC as a potential compensatory mechanism and target for social intervention is consistent with its known role in mentalizing, social cognition, and emotional processing. Dorsal medial PFC (dmPFC) has been shown to be involved in appraisal and expression of emotion while ventromedial PFC (vmPFC) regulates limbic regions involved in generating emotion [35]. The dmPFC has been considered a part of a “mentalizing network” [36, 37] and plays a critical role in self and other referential processing [38, 39]. The dmPFC is also known for playing a role in social cognition and has been found to preferentially respond to scenes of social interaction during viewing of a naturalistic movie [40]. The extent to which salience-enhancing intervention strategies are successful at engaging mPFC may vary depending on individual differences in social ability and sensory processing. For example, Green et al. [24] found that attentional direction engaged mPFC more for ASD youth with lower levels of SOR. In contrast, after attentional direction, higher SOR was associated with greater activation of disparate sensory cortical regions, suggesting a potentially less efficient strategy involving separately processing each individual social cue rather than integrating the information. Therefore, it is possible that explicit attentional direction may result in a more effortful, less efficient processing experience for youth with SOR who are already overwhelmed with interpreting multiple competing types of incoming information. To address this concern, we tested an implicit priming strategy—viewing one’s own emotion faces—to examine whether this could lead to more integrative, less effortful processing strategies.

In this study, we investigated how auditory distracters—common environmental noises—affect neural processing of emotional faces in youth with ASD and tested whether priming participants with videos of their own emotional expressions affects how they process others’ emotional faces*.* We hypothesized that we would see greater activation in the amygdala and decreased activation in face processing regions during trials with simultaneous auditory stimuli in the ASD group. We also expected that the effects of aversive auditory stimuli would be greater for youth with higher SOR. In addition, we predicted that the self-video prime would increase the salience of the social information and therefore attention to and understanding of others’ emotion faces, which would improve performance on the facial emotion recognition task in youth with ASD and engage areas involved in social cognition, especially mPFC. Finally, we hypothesized that, for youth with ASD, the effect of the prime on neural activation during emotion identification would differ depending on SOR severity.

## Methods

### Participants

Participants were 30 youth with ASD and 24 typically developing (TD) youth aged 8–17 years (*M* = 14.67; SD = 2.56). Participants were recruited based upon the presence or absence of an ASD diagnosis. Participants in the ASD group had a documented history of an ASD diagnosis which was confirmed for this study using a combination of the Autism Diagnostic Interview-Revised [41], the Autism Diagnostic Observation Schedule, 2nd Edition [42], and clinical judgment. FSIQ > 70 based on the Wechsler Abbreviated Scales of Intelligence 2nd Edition (WASI-II [43];), was an inclusion criterion for both groups. Participants were 33% White, 22% Hispanic or Latino/a, 22% Multiracial, 15% Asian, and 7% Black or African American (Table 1). The groups did not differ significantly in age, performance IQ, or motion during fMRI (Table 1). The TD group had significantly higher verbal and Full-scale IQ (FSIQ). Therefore, FSIQ was included as a covariate in all group comparisons. Data were originally acquired for 36 ASD and 33 TD subjects; 4 ASD and 7 TD participants were excluded due to excessive motion (mean absolute motion > 1 mm and max absolute > 4 mm). To reduce the chances that any pre- to post-prime differences were due to differences in motion, we also excluded any subjects who had a difference of > 0.1mm mean motion between the pre-prime and post-prime conditions (1 TD and 1 ASD). In addition, 1 ASD subject was excluded for having extremely low accuracy on the task (< 50% correct) and 1 TD subject was excluded for a self-video prompt with mismatched emotional valence which was a key component of the fMRI task paradigm (see “Emotion expressiveness”). No participants reported any neurological (e.g., epilepsy), genetic (e.g., Fragile X), or severe psychiatric disorder (e.g., schizophrenia) other than autism. Additionally, no TD participants had comorbid psychiatric disorders (e.g., ADHD, mood disorders, anxiety). Fourteen participants in the ASD group were taking psychoactive medications (selective serotonin reuptake inhibitors/selective serotonin-norepinephrine reuptake inhibitors, *N* = 3; psychostimulants, *N* = 1; centrally acting alpha-adrenergic receptor agonists, *N* = 1; multiple medications, *N* = 9). All parents provided written informed consent and youth gave written assent. Study procedures were approved by the UCLA Institutional Review Board.

**Table 1:** Descriptive statistics

	ASD (N=30)		TD (N=24)		***t*** or ***χ***2
	***N***	%	***N***	%	
**Gender (% male)**	21	70	15	62.5	0.34
**Race/ethnicity**					2.54
White, not Hispanic or Latino/a	9	30	9	37.5	
Asian, not Hispanic or Latino/a	3	10	5	20.83	
Black or African American, not Hispanic or Latino/a	3	10	1	4.17	
Hispanic or Latino/a	8	26. 67	4	16.67	
Multiracial, not Hispanic or Latino/a^1^	4	13.33	3	12.5	
Multiracial, Hispanic or Latino/a^2^	3	10	2	8.33	
	**Mean**	**SD**	**Mean**	**SD**	
**Age**	15.07	2.55	14.17	2.54	1.29
**VIQ**	104.53	17.11	115.08	12.11	− 2.50*
**FSIQ**	107.5	16.12	115.92	12.47	− 2.10*
**PIQ**	109.9	16.86	112.87	11.63	− 0.74
**Mean absolute motion**	0.45	0.2	0.36	0.23	1.52
**Mean relative motion**	0.13	0.05	0.15	0.17	− 0.46
**Mean volumes censored**	20.33	12.44	15.04	9.75	1.71^†^

*VIQ* Verbal IQ, *FSIQ* Full-scale IQ, *PIQ* Performance IQ

^†^*p* < 0.1, *p < 0.05

^1^6 participants identified as Asian & White; 1 identified as Black or African American & White

^2^1 participant identified as Hispanic or Latino/a, American Indian/Alaska Native, & White; 1 identified as Hispanic or Latino/a, Asian, & White; 1 identified as Hispanic or Latino/a, Native Hawaiian or Pacific Islander, Black or African American, & White; 1 identified as Hispanic or Latino/a, Asian, Black or African American, & White; 1 identified as Hispanic or Latino/a & Asian

### fMRI Paradigm

The emotion identification task was created using 72 faces from the NimStim Set of Facial Expressions [33, 44]: 36 males (24 Caucasian, 12 African American) and 36 females (18 Caucasian, 8 African American, 10 Asian). Each individual face trial lasted for 2200 ms and consisted of 20 separate images of the same individual’s face morphing from neutral to fully expressive (either happy or angry). The initial neutral image was shown for 300 ms to give participants time to orient to the stimulus and each subsequent image was shown for 100ms. Faces were presented such that the center of the screen was centered between the eyes. Inter-trial fixation intervals were pseudo-randomly jittered between 2000 and 3000 ms with a mean of 2500 ms. The task included 10 s of initial fixation and 8 s of final fixation. During the fixation intervals a cross was presented in the center of the screen. Stimuli were presented in four blocks of 18 face trials; during two of these blocks (50% of trials), each face trial was accompanied by simultaneous aversive environmental sounds (e.g., lawnmowers, police sirens, etc.). The race and gender of the faces were matched in each block of 18 trials. The noise stimuli were taken from a previously described paradigm where they were rated by an independent sample as moderately aversive on a 7-point Likert scale; root-mean-square amplitude was normalized across all stimuli to control for loudness [45]. After the first two blocks of the task, participants viewed two 19-second recorded videos of their own face making happy and angry expressions (see “Pre-task procedures”) followed by 2 s of rest. Then participants completed the last two blocks of the task. The stimulus presentation was counterbalanced such that participants heard the noises and saw different faces in different blocks. The counterbalanced orders were matched between diagnostic groups.

Prior to beginning the task, participants were instructed to “decide whether the faces you see are becoming happy or angry as quickly as you can” and respond using a button box. Accuracy and reaction time were recorded. Accuracy was indexed as the percentage of trials where a participant correctly identified the emotion of the face; missed trials were counted as incorrect in calculating accuracy. Reaction time was recorded as the time from the onset of the facial stimuli to the time that the participant pressed the button box; missed trials were excluded from reaction time calculations. Missed trials were not excluded from fMRI analysis as participants were still processing faces during these trials. Prior to starting the task, the examiner asked the participant to push the buttons indicating happy and angry to ensure their fingers were in the correct position and participants completed two practice trials inside the MRI scanner to ensure that they were correctly responding before starting the task. Due to a technical error, accuracy and reaction time data were not recorded for 1 TD participant; therefore, accuracy and reaction time analyses were conducted for 30 ASD and 23 TD participants.

### Pre-task procedures

Prior to the MRI scan, participants’ facial expressions were recorded while watching a series of video clips designed to elicit either happy or angry emotions. The negatively valanced clips were compiled from “Harry Potter and the Sorcerer’s Stone” (Warner Bros. Pictures, 2001), “Harry Potter and the Order of the Phoenix” (Warner Bros. Pictures, 2007), and “Matilda” (Jersey Films, 1996). The positively valanced clips were compiled from “Despicable Me” (Universal Pictures, 2010) and “The Boss Baby” (DreamWorks Animation, 2017). Prior to viewing each respective set of video clips, participants were instructed that they were going to see videos that often make people feel angry/happy and to try their best to react as naturally as possible. If the examiner did not see any appropriate emotional expression during the videos, they instructed the participant to look at the screen and gave a series of five prompts ranging from an attempt to elicit a more natural expression (i.e., “I’d like you to take a moment to think of something that makes you feel happy/angry. You can think about it to yourself. Ok got it? Now I want you to keep thinking about that thing that makes you happy/angry until I ask you to stop. Try to show on your face how you feel when you’re thinking about your happy/angry thing.”) to finally directing the participant to make a happy/angry face, only progressing to the next prompt if the participant still was not showing any emotion. Thirty participants (17 ASD, 13 TD) did not receive any additional prompts; 17 participants (10 ASD, 7 TD) received one prompt for either happy, angry, or both; 4 participants (1 ASD, 3 TD) received two prompts; 1 ASD participant received three prompts, and 1 ASD participant received all five prompts. The number of prompts given was not recorded for 1 TD participant. The diagnostic groups did not differ in the number of happy or angry prompts received (Happy: *X*^2^(4, 53) = 5.77, *p* = 0.22; Angry: *X*^2^(1, 53) = 0.45, *p* = 0.50). Videos were edited by a research assistant to include the most expressive 20 s for each emotion based upon the presence of smiles, frowns, and eyebrow movements (see “Emotion expressiveness”), repeating segments if necessary. Participants also completed two practice trials of the task outside of the scanner.

### fMRI data acquisition

Scans were acquired on a Siemens Prisma 3-T MRI scanner. Each functional run involved the acquisition of 554 multiband echo-planar imaging (EPI) volumes (gradient-echo, TR = 720 ms, TE = 37 ms, flip angle = 52°, 104 × 104 matrix, 208 mm FOV, 72 slices, voxel size = 2 × 2 × 2 mm). The Siemens “prescan normalize” option was used. Auditory stimuli were presented via magnet-compatible active noise-canceling headphones (Optoacoustics LTD) which were calibrated to specifically reduce the noise of the EPI sequence used. Participants wore earplugs to further reduce scanner noise. The volume settings on the stimulus computer and Optoacoustics headphones were standardized across participants and pilot testing was conducted to ensure the sounds were clearly audible and mildly to moderately aversive over the scanner noise. Faces were presented via magnet-compatible digital video goggles (Resonance Technology Inc). Stimuli were presented using E-Prime 2.0 Software [46] on a Dell Latitude E6430 laptop computer.

### Behavioral measures

Diagnostic and cognitive assessments were administered by a clinician or member of the research team. Sensory and anxiety questionnaires were completed by the parent about their child. Emotional expressiveness was coded by the research team.

### Sensory Processing 3-Dimensions Sensory Checklist

The Sensory Processing 3-Dimensions (SP-3D) Sensory Checklist [47] is a parent checklist of sensations that may bother their child that evolved from the Sensory Over-Responsivity (SenSOR) Scales. The number of items that parents rate as bothering their child has been shown to discriminate between children with and without SOR [47]. The auditory sensory over-responsivity subscale was used for this study.

### The Screen for Child Anxiety-Related Emotional Disorders Parent Version

The Screen for Child Anxiety-Related Emotional Disorders (SCARED) [48] is a 41-item parent report of child anxiety symptoms with good internal consistency, test-retest reliability, and discriminate validity. The total score was used as a continuous measure of anxiety symptom severity [48].

### Emotion expressiveness

To control for individual differences in emotion expressiveness, each participant’s emotion face video was coded after the visit on a scale from 0 to 3. A score of 0 indicated that the participant was displaying the incorrect emotional valence (i.e., appearing happy during the angry video). Only one participant had a score of 0 and that participant was excluded from further analyses. A score of 1 indicated the correct emotional valence but relatively flat (e.g., a small close-lipped smile or a stony stoic face), a score of 2 indicated more noticeable changes in the eyes, brows, and mouth to indicate the respective emotion, and a score of 3 indicated the most expressive individuals (e.g., full open-mouth smiles and laughter or clear prolonged furrowed brow and frowning). Total expressiveness scores for each participant were calculated by adding together scores for both the happy and angry videos. Each video was coded by a master coder (the study principal investigator) who was blind to the diagnostic group, and two to five additional study staff. Reliability was calculated such that exact agreement in scoring with the master coder was considered 100% reliable, a score 1 different from the master coder was considered 50% reliable, and greater differences were considered 0% reliable. Average reliability between staff coders and the master coder was 87% (range = 85–89%).

### fMRI data analysis

Analyses were performed using the FMRIB Software Library (FSL), version 5.0.11. Preprocessing included motion correction to the mean image, spatial smoothing (Gaussian kernel full width at half maximum = 5 mm), and high-pass temporal filtering (*t* > 0.01 Hz). Functional data were linearly registered to a common stereotaxic space by registering to the MNI152 T1 2-mm template (12 degrees of freedom). FSL’s fMRI Expert Analysis Tool (FEAT), version 6.0, was used for statistical analyses. Fixed-effects models were run separately for each subject then combined in a higher-level mixed-effects model to investigate within- and between-group differences. Single-subject models for all analyses included 12 motion parameters as covariates. Outlier motion volumes were identified using the FSL tool fsl_motion_outliers and were covaried out in the single-subject level analyses. For one participant, including motion parameters significantly increased motion artifact (i.e., motion ringing) due to task-correlated motion, so for this participant, only the motion outlier regressor was included. Each experimental condition (pre-prime no sound, pre-prime sound, post-prime no sound, post-prime sound collapsed across happy/angry trials) was modeled with respect to fixation during rest. Higher-level group analyses were carried out using FSL’s Local Analysis of Mixed Effects State (FLAME 1+2). Within-group activation maps were thresholded at *Z* > 3.1 and whole-brain cluster-corrected at *p* < 0.05. Between-group activation maps were thresholded at *Z* > 2.3 and whole-brain cluster-corrected at *p* < 0.05. After thresholding, within-group between-condition contrasts were masked by all voxels significant in each condition at *Z* > 1.7 for each group; between-group contrasts were then masked by all voxels significant in either diagnostic group for that contrast at *Z* > 2.3. Full-scale IQ and age were included as covariates in all analyses. Emotion expressiveness during the self-video and change in head motion from before to after the prime were tested as additional covariates to ensure they did not account for Prime > Pre-Prime change in blood oxygen level-dependent (BOLD) response, but neither covariate affected the Prime > Pre-Prime results so they were removed from these analyses.

### Correlation with auditory sensory over-responsivity

To determine whether the effects of the self-face video prime and auditory sensory distracters on brain response during emotion identification varied as a function of auditory SOR, regression analyses were performed with the auditory SOR subscale from the SP-3D Inventory entered as a regressor to predict change in BOLD response in a whole-brain analysis. These analyses were performed only within the ASD group as there was minimal variability in SOR in the TD group, with most participants scoring at or near the floor of the measure. Activation maps were thresholded at *Z* > 2.3 and whole-brain cluster corrected at *p* < 0.05. Full-scale IQ and age were included as covariates. Due to the high co-occurrence of SOR and anxiety, the total anxiety score from the SCARED was also included as a covariate to ensure the results observed were due to the effects of SOR over and above those of anxiety. Parameter estimates from these correlational whole-brain analyses were extracted from significant clusters to check that they were not driven by outliers; all correlations reported either had no significant outliers or survived with outliers removed.

## Results

### Behavioral results

As expected, the ASD group had significantly higher auditory SOR than the TD group (Table 2) with the ASD group mean falling above the cut-off of 4 for elevated sensory over-responsivity [4, 6, 47]. In addition, there was wide variability in auditory SOR within the ASD group (SD = 4.88, range = 0–16) in contrast to lower variability in the TD group (SD = 1.18, range = 0–5).

Independent samples *t*-tests showed that the ASD and TD groups did not differ significantly on overall accuracy or reaction time (Table 2). There were also no group differences in accuracy or reaction time in any individual condition (Table 2). Pearson correlations revealed that accuracy and reaction time were negatively correlated across the sample (*r* = − .64, *p* < 0.001) indicating that participants who were more accurate were also faster. Older participants also performed better on the task as age was correlated with accuracy (*r* = .45, *p* = 0.001) and negatively correlated with reaction time (*r* = − .33, *p* = 0.02). Accuracy and reaction time were not correlated with IQ or auditory SOR in either group (*r* ranging from .05 to .19, p>.10) with the exception of a trending correlation between accuracy and auditory SOR in the TD group (*r* = − .37, *p* = .08). However, this correlation was influenced by an outlier and was no longer trending when the outlier was removed (*r* = .07, *p* = .75). We tested for Group X Condition differences in accuracy and reaction time using a 2 × 2 repeated-measures ANOVA with Prime (Pre-Prime and Post-Prime) and Sound (Sound and No Sound) as within-group variables, Group (ASD or TD) as a between-group variable, and age and IQ as covariates. The accuracy analysis revealed no significant interactions or main effects. The reaction time analysis showed a non-significant trending Group X Sound interaction (*F*(1, 49) = 3.72, *p* = 0.06) indicating that the ASD participants trended towards faster reaction time on the trials with sound compared to TD as well as a trending Group X Prime interaction (*F*(1, 49) = 3.07, *p* = 0.09) indicating that the TD participants trended towards slower reaction times after the prime compared to ASD. There were no other significant or trending main effects or interactions.

An independent samples *t*-test revealed a trending difference in total expressiveness between the groups (Table 2) which was driven by a trending higher angry expressiveness score in the ASD group (Table 2). Across groups, age and total expressiveness were negatively correlated (*r* = − .40, *p* = 0.003) indicating that younger participants were more expressive in both diagnostic groups.

**Table 2:** Behavioral results

	ASD		TD		***t*** or ***χ***2
	Mean	SD	Mean	SD	
**Overall Accuracy**	0.90	0.07	0.89	0.08	0.16
**Overall Reaction Time (ms)**	1404	156.95	1425	166.77	− 0.48
**Pre-Prime No Sound Accuracy**	0.90	0.09	0.91	0.11	−0.35
**Pre-Prime Sound Accuracy**	0.91	0.09	0.89	0.11	0.76
**Post-Prime No Sound Accuracy**	0.90	0.11	0.89	0.12	0.21
**Post-Prime Sound Accuracy**	0.88	0.10	0.89	0.12	− 0.18
**Pre-Prime No Sound Reaction Time (ms)**	1455	178.8	1412	174.31	0.88
**Pre-Prime Sound Reaction Time (ms)**	1357	169.19	1392	177.66	− 0.73
**Post-Prime No Sound Reaction Time (ms)**	1444	189.53	1470	179.02	− 0.51
**Post-Prime Sound Reaction Time (ms)**	1367	195.37	1435	214.38	− 1.19
**Total Expressiveness Score**	4.27	1.29	3.67	1.2	1.75^†^
**Angry Expressiveness Score**	2.03	0.85	1.58	0.83	1.95^†^
**Happy Expressiveness Score**	2.23	0.73	2.08	0.78	0.73
**Sensory Processing 3-Dimensions (SP3D) Auditory Count**	4.5	4.88	0.58	1.18	4.24***

^†^*p* < 0.1, *p < 0.05, **p < 0.01, ***p < 0.001

### fMRI results

#### Pre-prime conditions within-group results

All within- and between-group results are listed in Tables 3-5, Additional File 1. While completing the task before the prime without sound present, both groups activated primary and secondary visual areas, insula, paracingulate gyrus, anterior cingulate cortex (ACC), thalamus, inferior frontal gyrus (IFG), and cerebellum (Supplementary Figure 1, Additional File 1). With the addition of sounds, both groups increased activation in the primary auditory cortex, IFG, and precuneus (Supplementary Figure 1, Additional File 1). There were no significant between-group differences in the Pre-Prime No Sound condition or in changes with the addition of sound.

#### Post-prime conditions within-group results

All within- and between-group results are listed in Tables 6-8, Additional File 1. When completing the task after seeing the self-face prime without sound, both groups activated many of the same regions observed before the prime, along with the ventromedial prefrontal cortex (vmPFC) in the ASD group (Supplementary Figure 2, Additional File 1). Both groups increased activity in the primary auditory cortex with the addition of sound (Supplementary Figure 2, Additional File 1). There were no significant between-group differences.

#### Sound > No Sound within-group results

**Fig. 1:**
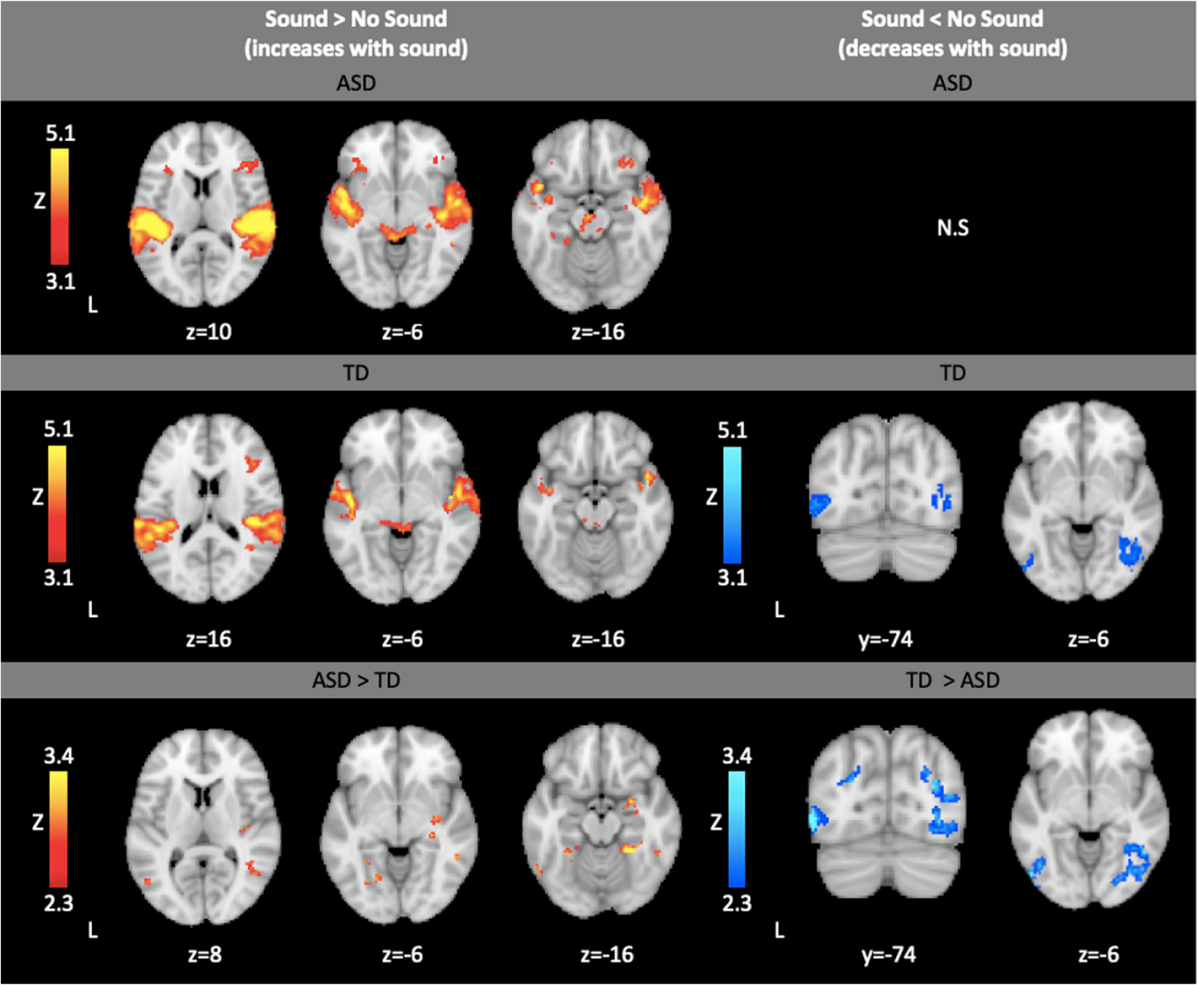
Within- and between-group results showing brain areas showing greater activation during trials with sound (Sound > No Sound) and within- and between-group results showing brain areas with lower activation during trials with sound (Sound < No Sound). Within-group contrasts thresholded at Z > 3.1, cluster-corrected (*p* < 0.05). Between-group contrasts thresholded at Z > 2.3, cluster-corrected (*p* < 0.05)

All within-group results are listed in Table 9, Additional File 1. Both groups had greater activation in bilateral Heschl’s gyrus and IFG during the trials with sound compared to those without sound (Fig. 1).

#### Sound > No Sound between-group results

All between-group results are listed in Table 9, Additional File 1. The ASD group showed greater increases than the TD group in the right amygdala, right putamen, hippocampus, insula, and a number of temporal and occipital regions (Fig. 1). In contrast, compared to the ASD group, the TD group showed greater decreases in activity in temporal and occipital regions including the fusiform cortex in the trials with sound compared to those without sound (Fig. 1).

#### Post-Prime > Pre-Prime within-group results

**Fig. 2:**
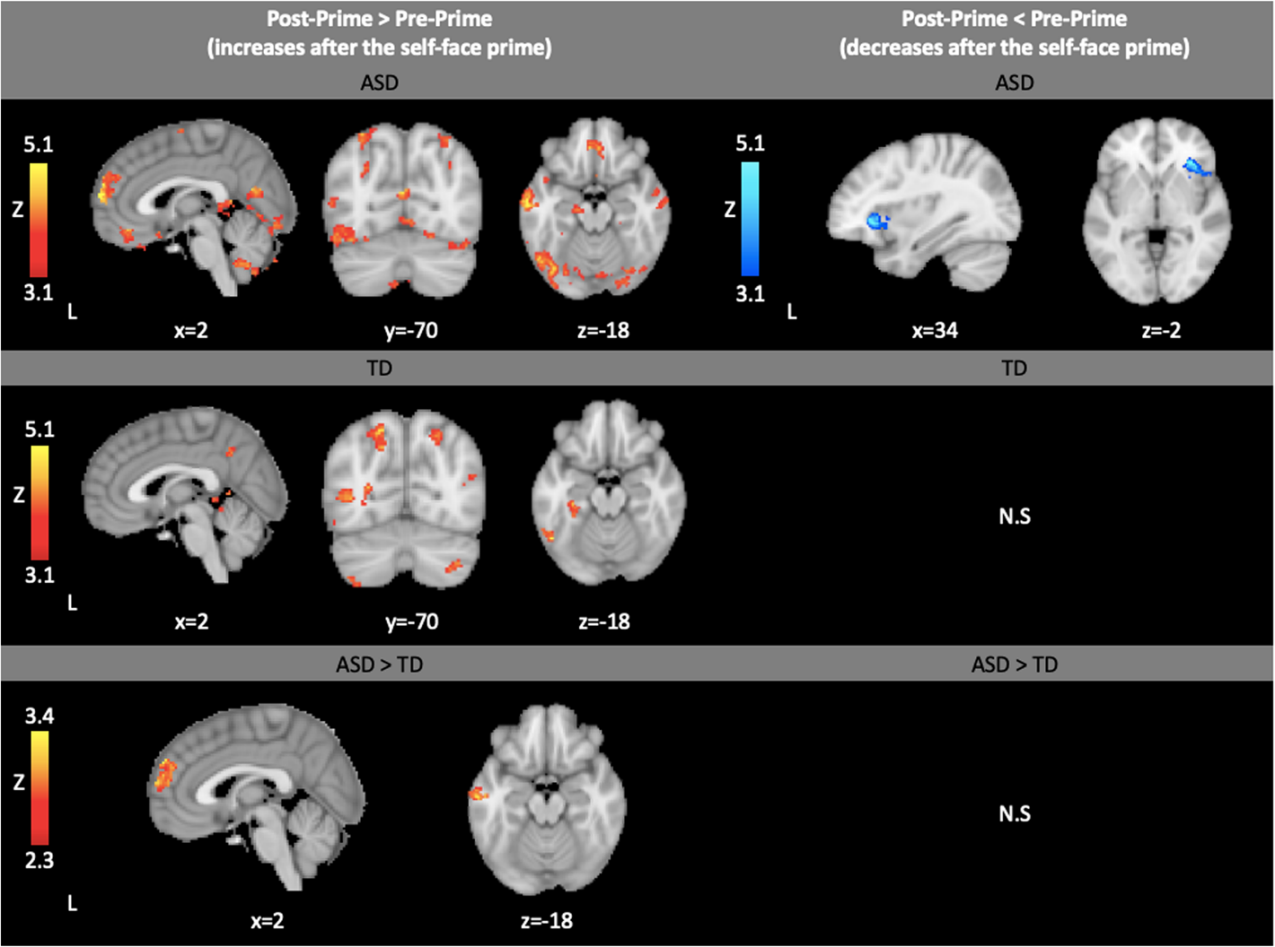
Within- and between-group results showing brain activation that increased after the self-face prime (Post-Prime > Pre-Prime) and within- and between-group results showing brain activation that decreased after the self-face prime (Post-Prime < Pre-Prime). Within-group contrasts thresholded at Z > 3.1, cluster-corrected (*p* < 0.05). Between-group contrasts thresholded at Z > 2.3, cluster-corrected (*p* < 0.05)

All within-group results listed in Table 10, Additional File 1. Both groups showed increases in occipital regions and hippocampus in responses after the prime compared to before the prime (Fig. 2). The ASD group also decreased activity in the right insula and operculum after the prime but did not show significantly different decreases in these regions compared to the TD group (Fig. 2).

#### Post-Prime > Pre-Prime between-group results

All between-group results are listed in Table 10, Additional File 1. The ASD group showed greater increases than the TD group in the medial prefrontal cortex (mPFC) and middle temporal gyrus (Fig. 2).

#### Correlations with auditory SOR severity

**Fig. 3:**
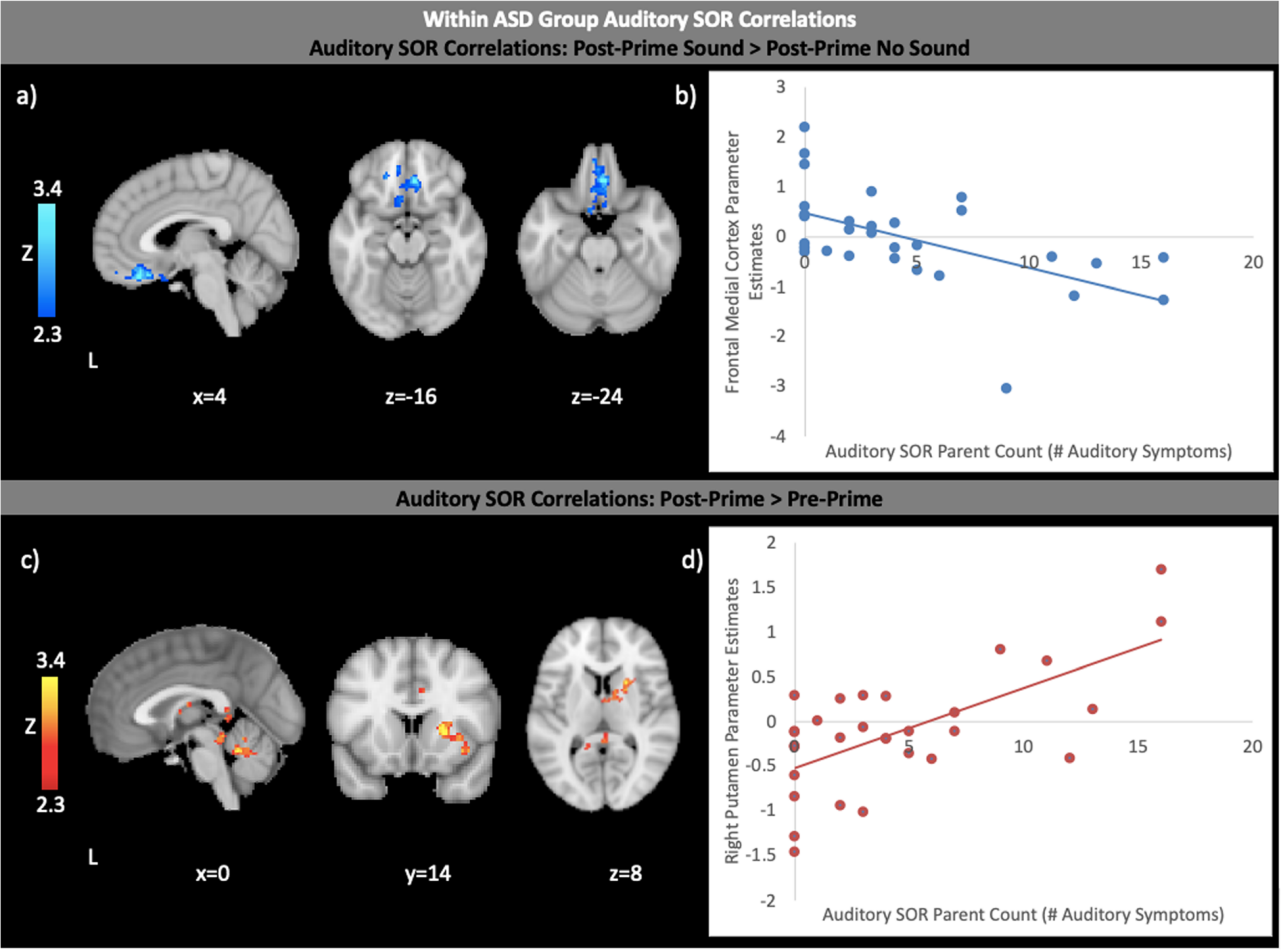
**a** Negative correlations (blue) with Auditory SOR within the ASD group in Post-Prime Sound > Post-Prime No Sound. Contrasts thresholded at Z > 2.3, cluster-corrected (*p* < 0.05). **b** Scatterplot illustrating a representative correlation between parent-reported count of Auditory SOR symptoms and brain responses within the ASD group. Horizontal axis: parent-reported count of Auditory SOR symptoms. Vertical axis: parameter estimates extracted from areas of Frontal Medial Cortex shown to correlate significantly with Auditory SOR. Correlations within all clusters remained significant after removal of potential outliers. All analyses covaried for Full-scale IQ, age, and SCARED total anxiety score. **c** Positive correlations (red) with Auditory SOR within the ASD group in Post-Prime > Pre-Prime. Contrasts thresholded at Z > 2.3, cluster-corrected (*p* < 0.05). **d** Scatterplot illustrating a representative correlation between parent-reported count of auditory SOR symptoms and brain responses within the ASD group. Horizontal axis: parent-reported count of auditory SOR symptoms. Vertical axis: parameter estimates extracted from areas of Right Putamen shown to correlate significantly with Auditory SOR. Correlations within all clusters remained significant after removal of potential outliers. All analyses covaried for Full-scale IQ, age, and SCARED total anxiety score

We examined how the severity of auditory SOR symptoms correlated with neural responses within the ASD group (Fig. 3; Supplementary Figure 3, Tables 11 and 12, Additional File 1). We did not examine SOR correlations within the TD group as these correlations would not be meaningful given the truncated range of SOR in this group, with most TD participants scoring at or near the floor of the measure. In the Pre-Prime No Sound condition, ASD youth with higher SOR had reduced activation in a number of temporal and subcortical regions (left amygdala, right putamen, right caudate, right accumbens) as well as right superior parietal lobule and postcentral gyrus (Supplementary Figure 3, Additional File 1). SOR did not correlate with any changes in brain response in the Pre-Prime Sound compared to Pre-Prime No Sound conditions (Supplementary Figure 3, Additional File 1). In the Post-Prime No Sound condition, youth with higher SOR had reduced activation only in the right superior parietal lobule and postcentral gyrus (Supplementary Figure 3, Additional File 1). After the Prime, youth with lower SOR showed greater increases in vmPFC activation in the Sound compared to No Sound condition (Fig. 3). We then examined how SOR correlated with changes in brain response after vs. before the prime, averaged across sound conditions (Fig. 3). Here, we found that higher SOR correlated with greater increases after the prime in the right thalamus, putamen, caudate, and insula, as well as the cerebellum. For representative scatterplots of neuroimaging correlational analyses, see Fig. 3.

## Discussion

To our knowledge, this is the first study to examine the neural effects of distracting sensory stimuli on emotion identification in youth with ASD. We additionally investigated the impact of a prime designed to increase the salience of the emotional cues by showing participants videos of their own faces making emotional expressions.

First, we found that there were no behavioral differences in accuracy or response time between ASD and TD youth during the emotion identification task, which indicates that both groups were able to understand and complete the task at a similar level of performance. Similarly, there were no group differences in the conditions where participants were identifying faces without background noise before seeing their own faces, potentially indicating that this is a relatively simple task that, when presented without distraction, can be easily accomplished by youth with ASD.

Secondly, we aimed to explore how distracting aversive background noises would affect brain responses while processing emotional faces in youth with ASD. With the addition of the auditory distracting stimuli, both groups showed similar increases in the auditory cortex and right IFG, a region that has been implicated in inhibition and attentional control [49]. This upregulation of IFG is also consistent with the pattern of results observed within the TD group in previous work investigating the effects of a simultaneous aversive tactile distracter during a social cognition task [24].

Group differences were seen in a number of regions when completing the task in the presence of aversive sounds: the ASD group showed greater increased activity compared to the TD group in limbic and salience regions, including the amygdala and insula, as well as primary sensory regions and fusiform. In contrast, the TD group showed decreased activity compared to the ASD group in the fusiform and primary visual cortex. It is possible that the ASD group was upregulating amygdala and fusiform activity, in order to complete the face processing task despite distraction, given the role of these structures in emotional face processing [50]. However, it is more likely that the increase in amygdala and insula activity reflects greater aversiveness and/or salience of the auditory stimuli in the ASD group given that these regions have previously been found to show over-reactivity in youth with ASD during aversive sensory stimulation even in the absence of a social task [27–29]. Additionally, the decrease in fusiform gyrus activity in the TD group is unexpected based on previous work showing that TD youth upregulate areas involved in language and social processing while completing a social cognition task in the presence of tactile distraction [24]. These different findings may be due to the fact that the emotional face processing task used in this study is a less complex social task that, unlike the previously used social inference task, did not require the integration of visual and auditory cues and therefore may be more automatic for the TD group. Our results showing that the ASD group displayed increased activity in lateral occipital cortex and primary visual regions when sound was added while the TD group showed decreased activity in these regions is consistent with previous work suggesting reduced differentiation and segregation of sensory systems in ASD [27, 51]. Overall, these group differences seen in neural face processing accompanied by auditory distraction are relevant for understanding how youth with ASD process noisy real-world environments that require multimodal sensory information processing.

Halfway through the task, participants were shown videos of their own faces making emotional expressions in an attempt to direct their attention towards relevant emotional cues expressed by others. After watching the self-video prime, both groups increased activation in visual processing areas when observing others’ emotional expressions, suggesting increased visual attention directed towards the faces, as well as in the hippocampus. The hippocampus plays a role in memory, including the recall of faces [52–55], suggesting that participants may be retrieving memories of their own emotion expressions while processing others’ emotions.

Significant group differences were also seen in changes in response after the self-video prime. The ASD group showed greater signal increases in dmPFC and middle temporal gyrus when completing the task compared to the TD group. These results indicate that the self-video prime was engaging prefrontal cortex regions involved in mentalizing and social cognition [36, 37] for the ASD group, potentially by increasing the salience of facial emotional expressions. This increase in mPFC is consistent with previous work demonstrating that directing attention to relevant social cues increased activity in mPFC for youth with ASD during a social cognition task [34] and during the same social cognition task in the presence of aversive tactile sensory distraction [24]. This similar pattern of results across studies using different social tasks, different modalities of sensory distraction, and different methods of directing attention, indicates that more general mechanisms may be involved when directing attention to relevant social cues and that this mechanism has the potential to be broadly targeted in the development of social skills interventions.

Finally, we investigated the relationship between auditory SOR within the ASD group and changes in brain activity during the task with the addition of sound and after viewing the self-video prime. While on average, all youth with ASD showed increased vmPFC activity following priming, youth with *lower* SOR specifically showed *greater* increases in vmPFC activation in post-prime trials with sound compared to no sound. The vmPFC plays a role in modulating emotional responses by regulating limbic structures such as the amygdala [35]. Therefore, while greater vmPFC involvement following the prime may help emotion regulation in the ASD group in general, regardless of SOR, youth with lower auditory SOR may be better able to sustain this vmPFC engagement in the context of distracting sound. These findings are consistent with other research suggesting that youth with lower SOR are better able to regulate their amygdala response to aversive sensory stimuli [27, 28]. ASD youth with lower SOR also showed greater activation of brain regions involved in emotion regulation and higher-level social cognition after their attention was directed to social cues while completing a social inference task with simultaneous tactile distraction [24]. Here we also found that higher SOR correlated with greater increased activity after the prime in brain regions related to salience detection and sensorimotor processing, including the insula, cerebellum, thalamus, and basal ganglia. As the basal ganglia are thought to be involved in the non-conscious perception of emotional signals [56], the prime may be recruiting these alternative pathways for processing emotional facial stimuli in youth with higher SOR. Higher SOR also correlated with greater post-prime signal increases in the thalamus, which is known for its role in modulating sensory input [57]. Previous research has shown that youth with ASD have reduced thalamocortical modulation during aversive sensory exposure [58] so it is possible that the prime may be helping high-SOR ASD youth filter the background noise and direct attention back towards the social stimuli through increased thalamic modulation. Importantly, while overall there were no overall diagnostic group differences in brain responses to faces prior to the prime, within the ASD group, youth with higher SOR showed reduced activation in a number of regions including amygdala and basal ganglia regions involved in salience detection and reward. In contrast, higher-SOR youth did not show reduced activation in these regions after the prime, which is consistent with the idea that the prime may have helped to increase salience and attention to others’ facial expressions. This finding that SOR moderates the effect of the prime on brain activation during a social task is consistent with our previous work showing the neural effects of explicit attentional direction to social cues differ based on SOR severity [24], and likely indicates that despite similar behavioral performance, high and low-SOR ASD youth maybe be using different neural strategies to complete the same task. However, in contrast to the Green et al. [24] study, in which high-SOR youth used a likely low-efficiency, high-effort strategy of processing multiple social cues separately (e.g., tone of voice, facial expression, visual background cues), here we found that SOR was associated with post-Prime increases in brain regions related to salience detection, non-conscious perception of emotional signals, and modulation of sensory inputs and attention. It is possible that the implicit nature of viewing one’s own emotional faces was better able to elicit subconscious, lower-effort modulation processes that helped to increase the relative salience of the social cues compared to the extraneous background noise in contrast with the explicit attentional direction used in the Green et al. [24] study. Our finding that high SOR was associated with reduced activation in salience detection and social reward regions before but not after the prime, further supports the idea that for this subset of youth with ASD, viewing one’s own emotional faces might increase the salience and intrinsic reward value of viewing others’ faces. Notably, both the explicit and implicit cues increased mPFC activation for lower-SOR youth, suggesting that the type of intervention may be less important for low-SOR youth who are less distracted by multiple, competing incoming stimuli.

Overall, the emotion identification task used in this study was designed to approximate a real-world multi-sensory environment by asking participants to complete a social task in the presence of common, mildly aversive environmental noises. However, the task was still over-simplified compared to real social interactions when face processing and emotion identification are more subtle and complex, which may explain why both groups showed similar, high levels of behavioral task performance in all conditions and did not show overall diagnostic group differences in neural activation before the prime. In addition, particularly given the heterogeneity within the ASD group, the task had reduced power to look at diagnostic group differences within each condition independently. The simplicity of the task limits our ability to fully interpret the success of the prime, which needs further study to examine its effectiveness (a) in more complex, real-world social scenarios, (b) when integrated into a larger-scale social skills intervention, and (c) across a longer period of time. For example, if the implicit priming strategy does indeed reduce the effort needed to process social stimuli, this would become more clear with more complex social situations, where task performance might improve, and over a longer period of time, where an extended, implicit, social-salience-enhancing intervention might reduce SOR-related challenges such as emotional and behavioral dysregulation or social anxiety. Despite these limitations, this study is an important preliminary proof-of-mechanism step showing that an implicit social prime can indeed target brain regions important to salience detection, enhancing social-emotional processing, and modulating sensory inputs. Taken together with prior research (e.g., [24]), this suggests that even very basic attentional direction strategies have an effect on neural responses to social cues for youth with ASD, particularly in the presence of distracting extraneous sensory stimuli.

An additional limitation is the challenge of examining responses to auditory stimuli in the noisy MRI environment. Pilot testing was conducted to ensure that the sounds were audible and aversive over the sounds of the scanner, and noise-canceling headphones helped reduce the sound of the scanner, but comparing one noisy condition to another (e.g., auditory environmental sounds with MRI sounds to MRI sounds only) is likely to reduce group differences in neural responses. Consistent with this, our previous studies have found fewer group differences in fMRI responses to auditory stimuli alone vs. tactile stimuli alone or simultaneous auditory and tactile stimuli [27–29]. Therefore, future studies might see greater group differences using a similar face processing task with a non-auditory modality of a sensory distractor.

Our task was also limited in that we were unable to conduct an additional control for potential order effects whereby neither group received the self-face prime. However, the most likely change we would expect due to order effects and time itself would be habituation and/or fatigue. The fact that we see greater activation in the Prime > Pre-Prime condition suggests that the prime is having an effect over and above habituation. In addition, an earlier behavioral study using this same task without auditory distractors tested for between-group effects of a self-face prime compared to an other-face prime, finding that only the self-face prime normalized reaction time for TD participants with high ASD traits [33]. This also suggests that the self-face prime is having an effect that is greater than pure timing effects. However, future studies with additional control groups who do not receive the self-face prime are needed to fully investigate this effect.

In addition, future research should investigate the effects of age upon both face processing and sensory over-responsivity in ASD. While our study took into account possible developmental effects by ensuring that groups were age-matched and covarying for age in all of our analyses, there is potential for future research to specifically examine these effects, especially given that younger participants were more expressive in their emotion videos used as the prime. Some of the differences seen in the face processing literature in ASD may be partially accounted for differing age of participants [11, 12], and one cross-sectional study indicated that functional connectivity of the fusiform face area correlates differently with age in ASD and TD groups [59]. Though not significant, the ASD group also trended towards being more expressive, which could relate to differences in developmental levels between the two groups. In addition, SOR has been found to decrease with age [60]. Therefore, future longitudinal research looking at the development of face processing, emotion expressiveness, and sensory processing in ASD and TD samples is needed to better understand the optimal target window for interventions.

## Conclusions

Taken together, our findings have implications for designing interventions for youth with ASD. First, we demonstrated that a simple prime designed to direct attention to appropriate social cues led to youth with ASD recruiting underlying neural circuitry involved in social cognition, even in the presence of aversive environmental noises. This indicates that more complex social interventions focused upon explicitly increasing salience of important social cues compared to extraneous sensory distracters might be able to tap into these same neural mechanisms. Second, we demonstrated that youth with ASD increased activity in limbic and salience regions when completing the task in the presence of aversive environmental noises. This suggests that the sensory environment should be taken into account when delivering a social intervention in order to maximize its effectiveness and translation into real-world settings, which are often noisy and filled with competing sensory information. Finally, our results showed that for youth with ASD, the effect of viewing their own emotional expressions on neural processing of others’ expressions varied depending on their auditory SOR levels. Based on this finding, the heterogeneity of sensory challenges in ASD should be taken into account as a part of treatment, even when designing interventions that target another domain such as social skills. For example, youth with ASD entering a social skills program could be screened for SOR to specifically practice skills with differing levels of sensory distraction, as well as coping strategies for managing SOR, when indicated. Overall, this research highlights the importance of taking a holistic approach to treating ASD symptomatology including both sensory and social components.

## Supplementary Information

**Additional file 1.**

## Data Availability

The dataset analyzed during the current study is available from the corresponding author on reasonable request.

## Abbreviations

ACC: Anterior cingulate cortex
ADHD: Attention deficit hyperactivity disorders
ASD: Autism spectrum disorders
BOLD: Blood oxygen level-dependent
DSM-IV: Diagnostic and Statistical Manual of Mental Disorders, 4th Edition
dmPFC: Dorsal medial prefrontal cortex
EEG: Electroencephalography
EPI: Echo-planar imaging
ERP: Event-related potentials
FEAT: FSL’s fMRI Expert Analysis Tool
FLAME: FSL’s Local Analysis of Mixed Effects State
fMRI: Functional magnetic resonance imaging
FSIQ: Full-scale IQ
FSL: FMRIB Software Library
IFG: Inferior frontal gyrus
mPFC: Medial prefrontal cortex
MRI: Magnetic resonance imaging
SCARED: Screen for Child Anxiety-Related Emotional Disorders
SenSOR: Sensory Over-Responsivity Scales
SOR: Sensory over-responsivity
SP-3D: Sensory Processing 3-Dimensions
SRS: Social Responsiveness Scale
TD: Typically developing
UCLA: University of California Los Angeles
VABS: Vineland Adaptive Behavior Scale
vmPFC: Ventromedial prefrontal cortex
