# Supplementary material for: Effects of sensory distraction and salience priming on emotion identification in autism: an fMRI study
Source: J Neurodev Disord. 2021 Sep 23;13:42. doi: 10.1186/s11689-021-09391-0 (PMC8461948; doi:10.1186/s11689-021-09391-0)
Supplement: Supplementary file 1 — Additional file 1. [file 11689_2021_9391_MOESM1_ESM.docx]

| Table 3. MNI Coordinates for Pre-Prime No Sound condition | | | | | | | | | | |
| --- | --- | --- | --- | --- | --- | --- | --- | --- | --- | --- |
|  | TD | | | | | ASD | | | | |
|  |  | MNI Peak (mm) | | | Max |  | MNI Peak (mm) | | | Max |
|  | Voxels | x | y | z | Z | Voxels | x | y | z | Z |
| **Visual Cortex (V1, Fusiform, Lateral Occipital Cortex)** | 12366 | -4 | -96 | 2 | 6.66 | 16357 | 48 | -80 | -10 | 8.43 |
| **Supplementary Motor Area/Paracingulate gyrus/Anterior Cingulate Cortex** | 750 | 8 | 8 | 50 | 6.37 | 820 | -4 | 14 | 46 | 4.85 |
| **Right Insula/Operculum/Inferior Frontal Gyrus** | 495 | 32 | 18 | 2 | 5.31 | 1982 | 48 | 16 | -2 | 7.15 |
| **Left Putamen** | 504 | -28 | 0 | 0 | 5.43 |  |  |  |  |  |
| Left Insula/Operculum |  | -36 | 0 | 4 | 4.68 | 481 | -40 | 20 | -2 | 5.43 |
| **Left Postcentral Gyrus** | 291 | -36 | -42 | 48 | 4.31 | 653 | -28 | -22 | 54 | 4.94 |
| **Left Precentral Gyrus** |  |  |  |  |  | 260 | -44 | 0 | 36 | 4.54 |
| **Right Thalamus (Ventral Anterior/Pulvinar Nuclei)** |  |  |  |  |  | 203 | 8 | -12 | 2 | 4.84 |
| **Left Thalamus (pulvinar nucleus)** | 184 | -20 | -30 | -4 | 4.42 |  |  |  |  |  |
| **Right Cerebellum (Lobule VIIb)** |  |  |  |  |  | 163 | 16 | -72 | -46 | 4.11 |
| **Right Superior Parietal Lobule** |  |  |  |  |  | 152 | 28 | -54 | 52 | 5.07 |
| **Right Putamen** | 130 | 20 | 8 | 6 | 4.92 |  |  |  |  |  |
| **Left Cerebellum** | 94 | -28 | -70 | -50 | 4.55 |  |  |  |  |  |
| Note: x, y, and z refer to the left-right, anterior-posterior, and inferior-superior dimensions; Z refers to the Z-score at those coordinates; voxels refers to the number of voxels in the cluster. Labels without voxels indicate submaxima within the same cluster as the label. Analyses thresholded at Z > 3.1, cluster-corrected (p<0.05). | | | | | | | | | | |

| Table 4. MNI Coordinates for Pre-Prime Sound condition | | | | | | | | | | | | | | | |
| --- | --- | --- | --- | --- | --- | --- | --- | --- | --- | --- | --- | --- | --- | --- | --- |
|  | TD | | | | | ASD | | | | | ASD > TD | | | | |
|  |  | MNI Peak (mm) | | | Max | | MNI Peak (mm) | | | Max |  | MNI Peak (mm) | | | Max |
|  | Voxels | x | y | z | Z | Voxels | x | y | z | Z | Voxels | x | y | z | Z |
| **Visual Cortex (V1, Fusiform, Lateral Occipital Cortex)** | 27074 | -20 | -74 | -10 | 8.7 | 37140 | 38 | -42 | -22 | 8.8 |  |  |  |  |  |
| Right Lateral Occipital Cortex |  | 32 | -82 | -14 | 6 |  | 42 | -82 | -14 | 7.7 | 382 | 42 | -70 | -2 | 3.7 |
| Left Auditory Cortex (Heschl's gyrus, superior temporal gyrus, planum polare) |  | -54 | -14 | -2 | 7.9 |  | -54 | -26 | 6 | 7.1 |  |  |  |  |  |
| Right Auditory Cortex (Heschl's gyrus, superior temporal gyrus, planum polare) |  | 40 | -26 | 14 | 6.4 |  | 60 | -20 | 10 | 6.7 |  |  |  |  |  |
| **Supplementary Motor Area/Paracingulate gyrus/Anterior Cingulate Cortex** | 1430 | 6 | 6 | 52 | 6.7 | 1364 | -6 | 14 | 38 | 6.8 |  |  |  |  |  |
| **Left Cerebellum (Lobule VIIb)** | 447 | -26 | -66 | -46 | 5.2 |  |  |  |  |  |  |  |  |  |  |
| **Left Putamen** |  |  |  |  |  | 360 | -18 | 6 | 8 | 4.7 |  |  |  |  |  |
| **Right Thalamus (Ventral Lateral Posterior Thalamic Nucleus, internal part)** | 114 | 12 | -16 | 8 | 5.2 |  |  |  |  |  |  |  |  |  |  |
| Note: x, y, and z refer to the left-right, anterior-posterior, and inferior-superior dimensions; Z refers to the Z-score at those coordinates; voxels refers to the number of voxels in the cluster. Labels without voxels indicate submaxima within the same cluster as the label. Analyses thresholded at Z > 3.1, cluster-corrected (p<0.05). | | | | | | | | | | |  |  |  |  |  |

| Table 5. MNI Coordinates for Pre-Prime Sound > Pre-Prime No Sound | | | | | | | | | | |
| --- | --- | --- | --- | --- | --- | --- | --- | --- | --- | --- |
|  | TD | | | | | ASD | | | | |
|  |  | MNI Peak (mm) | | | Max |  | MNI Peak (mm) | | | Max |
|  | Voxels | x | y | z | Z | Voxels | x | y | z | Z |
| **Right Auditory Cortex (Heschl's gyrus, superior temporal gyrus, planum polare)** | 4005 | 52 | -4 | -8 | 6.22 | 4997 | 56 | -28 | 10 | 7.42 |
| Right Inferior Frontal Gyrus | 387 | 42 | 26 | 16 | 4.94 |  | 46 | 30 | 8 | 4.21 |
| **Left Insula** |  |  |  |  |  | 4370 | -38 | -4 | -12 | 6.99 |
| Left Auditory Cortex (Heschl's gyrus, superior temporal gyrus, planum polare) | 4250 | -46 | -22 | -6 | 7.37 |  | -40 | -28 | 10 | 6.7 |
| **Cerebellum (Left Crus II)** | 389 | -18 | -84 | -34 | 5.49 | 341 | -12 | -84 | -34 | 5.43 |
| **Left Orbital Frontal Cortex** |  |  |  |  |  | 372 | -36 | 28 | -6 | 4.79 |
| **Brainstem** | 145 | 4 | -38 | -6 | 4.71 | 148 | 8 | -34 | -8 | 4.73 |
| **Left Inferior Frontal Gyrus** | 134 | -44 | 22 | 14 | 4.26 | 111 | -44 | 18 | 14 | 3.83 |
| **Cerebellum (Right Crus II)** | 78 | 10 | -88 | -40 | 4.82 | 127 | 16 | -78 | -34 | 4.02 |
| **Left Middle Frontal Gyrus** | 101 | -36 | 8 | 38 | 4.6 |  |  |  |  |  |
| **Right Orbital Frontal Cortex** | 92 | 34 | 28 | -16 | 4.11 |  |  |  |  |  |
| **Posterior Cingulate Gyrus/Precuneous/Lingual Gyrus** | 40 | -6 | -52 | 4 | 4.56 | 51 | 14 | -48 | 4 | 4.3 |
| **Right Frontal Pole** | 28 | 20 | 50 | -12 | 4.0 |  |  |  |  |  |
| **Left Middle Temporal Gyrus** | 25 | -54 | -48 | -6 | 4.17 |  |  |  |  |  |
| **Left Hippocampus** |  |  |  |  |  | 23 | -10 | -40 | 2 | 4.36 |
| **Cerebellum (Right Crus I)** | 11 | 36 | -80 | -30 | 3.86 |  |  |  |  |  |
| Note: x, y, and z refer to the left-right, anterior-posterior, and inferior-superior dimensions; Z refers to the Z-score at those coordinates; voxels refers to the number of voxels in the cluster. Labels without voxels indicate submaxima within the same cluster as the label. Analyses thresholded at Z > 3.1, cluster-corrected (p<0.05). | | | | | | | | | | |

| Table 6. MNI Coordinates for Post-Prime No Sound condition | | | | | | | | | | |
| --- | --- | --- | --- | --- | --- | --- | --- | --- | --- | --- |
|  | TD | | | | | ASD | | | | |
|  |  | MNI Peak (mm) | | | Max |  | MNI Peak (mm) | | | Max |
|  | Voxels | x | y | z | Z | Voxels | x | y | z | Z |
| **Visual Cortex (V1, Fusiform, Lateral Occipital Cortex)** | 17887 | -44 | -44 | -12 | 7.71 | 21162 | 40 | -42 | -18 | 8.02 |
| **Left Putamen/Amygdala** | 5924 | -28 | -2 | -4 | 6.72 |  |  |  |  |  |
| Left Thalamus (Lateral Geniculate Nucleus) |  | -20 | -30 | -2 | 4.82 | 294 | -22 | -28 | -6 | 4.45 |
| Left Precentral Gyrus |  | -54 | 8 | 22 | 6.2 | 2225 | -44 | 4 | 38 | 5.86 |
| **Supplementary Motor Area/Paracingulate gyrus/Anterior Cingulate Cortex** | 1472 | 4 | 8 | 54 | 7.56 | 1542 | 6 | 14 | 44 | 8.34 |
| **Right Precentral gyrus/Inferior Frontal Gyrus** |  |  |  |  |  | 1237 | 50 | 0 | 38 | 5.65 |
| Right Inferior Frontal Gyrus, Pars Opercularis | 1034 | 46 | 6 | 18 | 4.82 |  | 48 | 16 | 22 | 4.86 |
| Right Insula/Frontal Operculum |  |  |  |  |  |  | 46 | 12 | -2 | 5.23 |
| **Right Cerebellum (Lobule VIIb)** | 1083 | 18 | -68 | -44 | 6.17 |  |  |  |  |  |
| **Left Cerebellum (Left Lobule VIIb)** | 594 | -30 | -68 | -52 | 6.99 |  |  |  |  |  |
| **Right Amygdala/Putamen** | 379 | 26 | -2 | -14 | 5.21 |  |  |  |  |  |
| **Right Superior Parietal Lobule** | 179 | 28 | -48 | 50 | 5.24 |  |  |  |  |  |
| **Ventromedial Prefrontal Cortex** |  |  |  |  |  | 126 | 2 | 34 | -26 | 4.96 |
| **Right Thalamus (Lateral Geniculate Nucleus)** | 124 | 24 | -26 | -8 | 4.77 |  |  |  |  |  |
| **Left Insula** |  |  |  |  |  | 117 | -36 | 22 | -2 | 4.71 |
| **Right Temporal Pole** |  |  |  |  |  | 100 | 40 | 22 | -23 | 4.62 |
| **Right Parahippocampal Gyrus/Fusiform Gyrus** | 97 | 32 | -4 | -34 | 4.5 |  |  |  |  |  |
| **Right Inferior Frontal Gyrus, Pars Triangularis** | 94 | 46 | 34 | 0 | 4.66 |  |  |  |  |  |
| Note: x, y, and z refer to the left-right, anterior-posterior, and inferior-superior dimensions; Z refers to the Z-score at those coordinates; voxels refers to the number of voxels in the cluster. Labels without voxels indicate submaxima within the same cluster as the label. Analyses thresholded at Z > 3.1, cluster-corrected (p<0.05). | | | | | | | | | |  |

| Table 7. MNI Coordinates for Post-Prime Sound condition | | | | | | | | | | |
| --- | --- | --- | --- | --- | --- | --- | --- | --- | --- | --- |
|  | TD | | | | | ASD | | | | |
|  |  | MNI Peak (mm) | | | Max |  | MNI Peak (mm) | | | Max |
|  | Voxels | x | y | z | Z | Voxels | x | y | z | Z |
| **Visual Cortex (V1, Fusiform, Lateral Occipital Cortex)** | 22694 | -2 | -88 | -10 | 7.12 | 38070 | -20 | -76 | -12 | 8.13 |
| Left Auditory Cortex (Heschl's gyrus, superior temporal gyrus, planum polare) |  | -54 | -18 | 6 | 5.65 |  | -46 | -16 | 6 | 7.02 |
| Right Auditory Cortex (Heschl's gyrus, superior temporal gyrus, planum polare) |  | 50 | -18 | 8 | 5.93 |  | 48 | -14 | 6 | 7.36 |
| Right Precentral Gyrus | 355 | 36 | 6 | 28 | 4.1 |  | 50 | 4 | 36 | 5.24 |
| **Paracingulate gyrus/Anterior Cingulate Cortex** |  |  |  |  |  | 1470 | 4 | 14 | 44 | 5.85 |
| Supplementary Motor Area | 453 | 0 | -2 | 58 | 4.79 |  | -6 | 4 | 54 | 5.07 |
| **Left Postcentral Gyrus** | 568 | -46 | -32 | 54 | 4.63 | 1423 | -32 | -30 | 46 | 5.65 |
| Left Precentral Gyrus |  | -38 | -22 | 60 | 4.01 | 1228 | -42 | 0 | 34 | 6.17 |
| **Left Inferior Frontal Gyrus, Pars Opercularis** | 789 | -48 | 16 | 22 | 6 |  |  |  |  |  |
| **Right Inferior Frontal Gyrus, Pars Triangularis** | 295 | 48 | 32 | 2 | 4.63 |  |  |  |  |  |
| **Left Putamen** | 234 | -28 | -8 | -6 | 4.41 |  |  |  |  |  |
| **Left Thalamus (Lateral Pulvinar Nucleus)** | 216 | -20 | -32 | -2 | 4.75 |  |  |  |  |  |
| **Right Temporal Pole** |  |  |  |  |  | 198 | 42 | 20 | -22 | 4.73 |
| Ventromedial Prefrontal Cortex |  |  |  |  |  | 129 | 4 | 40 | -20 | 4.87 |
| **Left Frontal Operculum/Inferior Frontal Gyrus, Pars Triangularis** | 122 | -46 | 28 | 0 | 4.2 |  |  |  |  |  |
| **Superior Frontal Gyrus (Dorsal Medial Prefrontal Cortex)** |  |  |  |  |  | 114 | 6 | 58 | 22 | 4.76 |
| Note: x, y, and z refer to the left-right, anterior-posterior, and inferior-superior dimensions; Z refers to the Z-score at those coordinates; voxels refers to the number of voxels in the cluster. Labels without voxels indicate submaxima within the same cluster as the label. Analyses thresholded at Z > 3.1, cluster-corrected (p<0.05). | | | | | | | | | | |

| Table 8. MNI Coordinates for Post-Prime Sound > Post-Prime No Sound | | | | | | | | | | |
| --- | --- | --- | --- | --- | --- | --- | --- | --- | --- | --- |
|  | TD | | | | | ASD | | | | |
|  |  | MNI Peak (mm) | | | Max |  | MNI Peak (mm) | | | Max |
|  | Voxels | x | y | z | Z | Voxels | x | y | z | Z |
| **Right Auditory Cortex (Heschl's gyrus, superior temporal gyrus, planum polare)** | 1799 | 54 | -14 | 4 | 6.49 | 3566 | 66 | -28 | 10 | 7.2 |
| **Left Auditory Cortex (Heschl's gyrus, superior temporal gyrus, planum polare)** | 1556 | -38 | -30 | 8 | 6.62 | 3242 | -44 | -26 | 8 | 7.2 |
| Note: x, y, and z refer to the left-right, anterior-posterior, and inferior-superior dimensions; Z refers to the Z-score at those coordinates; voxels refers to the number of voxels in the cluster. Analyses thresholded at Z > 3.1, cluster-corrected (p<0.05). | | | | | | | | | | |

| Table 9. MNI Coordinates for Sound > No Sound and No Sound > Sound | | | | | | | | | | | | | | | |
| --- | --- | --- | --- | --- | --- | --- | --- | --- | --- | --- | --- | --- | --- | --- | --- |
| **Sound > No Sound** | | | | | | | | | | | | | | | |
|  | TD | | | | | ASD | | | | | ASD > TD | | | | |
|  |  | MNI Peak (mm) | | | Max |  | MNI Peak (mm) | | | Max |  | MNI Peak (mm) | | | Max |
|  | Voxels | x | y | z | Z | Voxels | x | y | z | Z | Voxels | x | y | z | Z |
| **Right Auditory Cortex (Heschl's gyrus, superior temporal gyrus, planum polare)** | 3441 | 46 | -20 | 6 | 6.78 | 5720 | 66 | -28 | 10 | 7.27 |  |  |  |  |  |
| **Left Auditory Cortex (Heschl's gyrus, superior temporal gyrus, planum polare)** | 3302 | -54 | -18 | 4 | 7.09 | 5077 | -40 | -28 | 10 | 7.68 |  |  |  |  |  |
| **Left Cerebellum (Left Crus II)** |  |  |  |  |  | 1565 | -6 | -82 | -34 | 6.3 |  |  |  |  |  |
| **Brainstem/Superior Colliculus** | 264 | 10 | -34 | -12 | 5.6 | 587 | -4 | -38 | -8 | 5.4 |  |  |  |  |  |
| **Orbital Frontal Cortex** |  |  |  |  |  | 426 | 30 | 30 | -20 | 4.57 |  |  |  |  |  |
| Right Inferior Frontal Gyrus | 94 | 42 | 24 | 16 | 4.62 |  | 46 | 32 | 10 | 3.88 |  |  |  |  |  |
| **Right Inferior Temporal Gyrus** |  |  |  |  |  |  |  |  |  |  | 238 | 48 | -44 | -12 | 3.86 |
| **Left Cerebellum (Lobule VI)** |  |  |  |  |  | 175 | -28 | -34 | -38 | 4.87 |  |  |  |  |  |
| **Right Cerebellum (Lobule VI)** |  |  |  |  |  | 156 | 28 | -38 | -38 | 4.71 |  |  |  |  |  |
| **Right Amygdala** |  |  |  |  |  |  |  |  |  |  | 142 | 26 | 0 | -16 | 3.52 |
| Right Putamen |  |  |  |  |  |  |  |  |  |  |  | 32 | -8 | -14 | 2.69 |
| **Left Inferior Frontal Gyrus, pars triangularis** |  |  |  |  |  | 122 | -44 | 28 | 18 | 4.51 |  |  |  |  |  |
| **Right Fusiform** |  |  |  |  |  |  |  |  |  |  | 123 | 28 | -44 | -18 | 3.98 |
| **Left Lateral Occipital Cortex, Superior Division/Precuneus** |  |  |  |  |  |  |  |  |  |  | 73 | -20 | -84 | 22 | 3.46 |
| **Right Lingual Gyrus** |  |  |  |  |  |  |  |  |  |  | 50 | 16 | -52 | 2 | 3.12 |
| **Left Lingual Gyrus** |  |  |  |  |  | 50 | -18 | -54 | 2 | 4.21 | 31 | -20 | -64 | -4 | 3.52 |
| **Left Lateral Occipital Cortex, Inferior Division** |  |  |  |  |  |  |  |  |  |  | 48 | -50 | -70 | 0 | 3.68 |
| **Parietooccipital Sulcus** |  |  |  |  |  |  |  |  |  |  | 42 | 16 | -84 | 46 | 3.2 |
| **Right Cerebellum (Right Crus II)** | 30 | 14 | -86 | -40 | 4.13 |  |  |  |  |  |  |  |  |  |  |
| **Primary Visual Cortex** |  |  |  |  |  |  |  |  |  |  | 26 | 0 | -86 | 30 | 3.31 |
| **Left Fusiform Gyrus** |  |  |  |  |  |  |  |  |  |  | 25 | -30 | -44 | -18 | 3.44 |
| **Left Parahippocampal Gyrus** |  |  |  |  |  | 22 | -20 | -38 | -18 | 3.97 |  |  |  |  |  |
| **Right Insula** |  |  |  |  |  |  |  |  |  |  | 21 | 32 | 10 | -8 | 3.31 |
| **Left Cerebellum (Lobule VI)** |  |  |  |  |  |  |  |  |  |  | 20 | -28 | -56 | -20 | 2.95 |
| **Left Lateral Occipital Cortex/Inferior Temporal Gyrus** |  |  |  |  |  |  |  |  |  |  | 19 | -54 | -68 | -10 | 3.36 |
| **Right Cerebellum (Right Crus I)** |  |  |  |  |  |  |  |  |  |  | 16 | 40 | -58 | -26 | 3.02 |
| **No Sound > Sound** | | | | | | | | | | | | | | | |
|  | TD | | | | | ASD | | | | | TD > ASD | | | | |
|  |  | MNI Peak (mm) | | | Max |  | MNI Peak (mm) | | | Max |  | MNI Peak (mm) | | | Max |
|  | Voxels | x | y | z | Z | Voxels | x | y | z | Z | Voxels | x | y | z | Z |
| **Right Inferior Temporal Gyrus/Lateral Occipital Cortex** | 500 | 48 | -56 | -4 | 5.11 |  |  |  |  |  | 1330 | 48 | -46 | -12 | 4 |
| **Left Lateral Occipital Cortex/Inferior Temporal Gyrus** |  |  |  |  |  |  |  |  |  |  | 536 | -52 | -74 | -8 | 4.24 |
| **Right Lateral Occipital Cortex** | 247 | -52 | -76 | -2 | 4.97 |  |  |  |  |  |  |  |  |  |  |
| **Left Lateral Occipital Cortex, superior division** |  |  |  |  |  |  |  |  |  |  | 215 | -24 | -78 | 26 | 3.55 |
| Note: x, y, and z refer to the left-right, anterior-posterior, and inferior-superior dimensions; Z refers to the Z-score at those coordinates; voxels refers to the number of voxels in the cluster. Labels without voxels indicate submaxima within the same cluster as the label. Within-group analyses thresholded at Z > 3.1, cluster-corrected (p<0.05). Between-group analyses thresholded at Z > 2.3, cluster-corrected (p<0.05). | | | | | | | | | | | | | | | |

| Table 10. MNI Coordinates for Post-Prime > Pre-Prime and Pre-Prime > Post-Prime | | | | | | | | | | | | | | | |
| --- | --- | --- | --- | --- | --- | --- | --- | --- | --- | --- | --- | --- | --- | --- | --- |
| **Post-Prime > Pre-Prime** | | | | | | | | | | | | | | | |
|  | TD | | | | | ASD | | | | | ASD > TD | | | | |
|  |  | MNI Peak (mm) | | | Max |  | MNI Peak (mm) | | | Max |  | MNI Peak (mm) | | | Max |
|  | Voxels | x | y | z | Z | Voxels | x | y | z | Z | Voxels | x | y | z | Z |
| **Primary Visual Cortex (V1)** |  |  |  |  |  | 929 | 0 | -70 | 16 | 5.03 |  |  |  |  |  |
| **Left Lateral Occipital Cortex** | 421 | -40 | -68 | 6 | 4.8 | 872 | -40 | -80 | -10 | 4.97 |  |  |  |  |  |
| **Dorsal Medial Prefrontal Cortex** |  |  |  |  |  | 514 | 2 | 64 | 14 | 6.01 | 389 | 4 | 58 | 30 | 3.75 |
| **Left Cerebellum (Lobule IX)** |  |  |  |  |  | 344 | -6 | -46 | -44 | 5.23 |  |  |  |  |  |
| **Left Inferior Temporal Gyrus** | 332 | -50 | -58 | -18 | 5.2 |  |  |  |  |  |  |  |  |  |  |
| **Left Middle Temporal Gyrus** |  |  |  |  |  | 315 | -68 | -18 | -12 | 5.6 | 167 | -60 | -14 | -18 | 3.9 |
| **Left Lateral Occipital Cortex, Superior Division** | 256 | -16 | -72 | 52 | 5.1 | 260 | -30 | -70 | 58 | 4.59 |  |  |  |  |  |
| **Ventromedial Prefrontal Cortex** |  |  |  |  |  | 198 | -2 | 40 | -26 | 5.27 |  |  |  |  |  |
| **Right Middle Temporal Gyrus** |  |  |  |  |  | 195 | 64 | -16 | -22 | 4.17 |  |  |  |  |  |
| **Right Lateral Occipital Cortex** | 181 | 46 | -78 | 14 | 4.6 | 150 | 24 | -76 | 50 | 4.8 |  |  |  |  |  |
| **Left Hippocampus/Left Parahippocampal Gyrus** | 77 | -32 | -26 | -18 | 4.6 | 177 | -34 | -28 | -10 | 5.47 |  |  |  |  |  |
| **Left Cerebellum (Left Crus II)** | 95 | -34 | -76 | -52 | 4.5 |  |  |  |  |  |  |  |  |  |  |
| **Left Inferior Frontal Gyrus/Frontal Pole** |  |  |  |  |  | 91 | -48 | 42 | 2 | 3.95 |  |  |  |  |  |
| **Right Lateral Occipital Cortex, Superior Division** | 82 | 22 | -70 | 48 | 4.2 |  | 56 | -62 | 22 | 3.84 |  |  |  |  |  |
| **Right Parahippocampal Gyrus/Hippocampus** |  |  |  |  |  | 79 | 20 | -26 | -14 | 4.29 |  |  |  |  |  |
| **Right Cerebellum (Right Crus II)** | 78 | 32 | -72 | -46 | 4.3 |  |  |  |  |  |  |  |  |  |  |
| **Left Temporal Pole** |  |  |  |  |  | 76 | -36 | 14 | -24 | 4.08 |  |  |  |  |  |
| **Left Postcentral Gyrus** | 74 | -28 | -36 | 70 | 4.8 |  |  |  |  |  |  |  |  |  |  |
| **Left Superior Frontal Gyrus** |  |  |  |  |  | 46 | -14 | 48 | 34 | 4.96 |  |  |  |  |  |
| **Right Thalamus (Pulvinar Nucleus)** | 11 | 8 | -30 | 12 | 4.4 | 32 | 22 | -34 | 4 | 4.32 |  |  |  |  |  |
| **Left Supplementary Motor Area** |  |  |  |  |  | 28 | -2 | -10 | 70 | 4.11 |  |  |  |  |  |
| **Precuneus** | 28 | 2 | -54 | 38 | 4.1 |  |  |  |  |  |  |  |  |  |  |
| **Right Superior Frontal Gyrus** |  |  |  |  |  | 16 | 16 | 46 | 34 | 3.93 |  |  |  |  |  |
| **Frontal Pole** |  |  |  |  |  | 14 | -24 | 52 | 6 | 4.08 |  |  |  |  |  |
| **Subcallosal cortex** |  |  |  |  |  | 12 | 4 | 12 | -22 | 4.17 |  |  |  |  |  |
| **Left Fusiform** |  |  |  |  |  | 12 | -32 | -38 | -22 | 3.64 |  |  |  |  |  |
| **Pre-Prime > Post Prime** | | | | | | | | | | | | | | | |
|  | TD | | | | | ASD | | | | | ASD > TD | | | | |
|  |  | MNI Peak (mm) | | | Max |  | MNI Peak (mm) | | | Max |  | MNI Peak (mm) | | | Max |
|  | Voxels | x | y | z | Z | Voxels | x | y | z | Z | Voxels | x | y | z | Z |
| **Right Insula/Operculum** |  |  |  |  |  | 267 | 32 | 30 | 0 | 6.36 |  |  |  |  |  |
| Note: x, y, and z refer to the left-right, anterior-posterior, and inferior-superior dimensions; Z refers to the Z-score at those coordinates; voxels refers to the number of voxels in the cluster. Labels without voxels indicate submaxima within the same cluster as the label. Within-group analyses thresholded at Z > 3.1, cluster-corrected (p<0.05). Between-group analyses thresholded at Z > 2.3, cluster-corrected (p<0.05). | | | | | | | | | | | | | | | |

| Table 11. MNI Coordinates for areas negatively correlated with Auditory SOR within ASD Group | | | | | | | | | | | | | | | | | | | | | | | | |
| --- | --- | --- | --- | --- | --- | --- | --- | --- | --- | --- | --- | --- | --- | --- | --- | --- | --- | --- | --- | --- | --- | --- | --- | --- |
|  | Pre-Prime No Sound Condition | | | | | Pre-Prime Sound Condition | | | | | | Post-Prime No Sound Condition | | | | | | | | Post-Prime Sound > Post-Prime No Sound | | | | |
|  |  | MNI Peak (mm) | | | Max |  | MNI Peak (mm) | | | Max | |  | | MNI Peak (mm) | | | | | Max |  | MNI Peak (mm) | | | Max |
|  | Voxels | x | y | z | Z | Voxels | x | y | z | Z | | Voxels | | x | | y | | z | Z | Voxels | x | y | z | Z |
| **Left Inferior Temporal Gyrus** | 1453 | -54 | -54 | -26 | 3.94 |  |  |  | | |  | |  |  |  | |  |  |  |  |  |  |  |  |
| **Right Cerebellum (Right Crus II)** | 1270 | 16 | -82 | -40 | 4.37 |  |  |  |  |  | |  | |  | |  | |  |  |  |  |  |  |  |
| **Left Cerebellum (Left Crus II)** |  |  |  |  |  | 1236 | -32 | -68 | -44 | 3.67 | |  | |  | |  | |  |  |  |  |  |  |  |
| **Left Cerebellum (Left Lobule I-IV/Lobule V)** | 791 | -12 | -44 | -12 | 3.78 |  |  |  |  |  | |  | |  | |  | |  |  |  |  |  |  |  |
| **Left Middle Temporal Gyrus/Temporal pole** | 688 | -48 | 0 | -26 | 3.78 |  |  |  |  |  | |  | |  | |  | |  |  |  |  |  |  |  |
| **Right Postcentral Gyrus/Superior Parietal Lobule** | 675 | 24 | -38 | 56 | 3.85 | 545 | 32 | -44 | 56 | 3.4 | | 375 | | 32 | | -56 | | 66 | 3.5 |  |  |  |  |  |
| **Brainstem** |  |  |  |  |  | 592 | -10 | -26 | -16 | 3.83 | |  | |  | |  | |  |  |  |  |  |  |  |
| Left Hippocampus |  |  |  |  |  |  | -28 | -16 | -16 | 3.15 | |  | |  | |  | |  |  |  |  |  |  |  |
| **Subcallosal Cortex/Ventromedial Prefrontal Cortex** | 356 | -8 | 26 | -28 | 3.52 |  |  |  |  |  | |  | |  | |  | |  |  | 612 | -6 | 8 | -26 | 4.06 |
| Left Amygdala |  | -20 | -2 | -12 | 3.5 |  |  |  |  |  | |  | |  | |  | |  |  |  |  |  |  |  |
| **Right Caudate/Accumbens/Putamen** | 343 | 10 | 14 | -2 | 4.04 |  |  |  |  |  | |  | |  | |  | |  |  |  |  |  |  |  |
| Note: x, y, and z refer to the left-right, anterior-posterior, and inferior-superior dimensions; Z refers to the Z-score at those coordinates; voxels refers to the number of voxels in the cluster. Labels without voxels indicate submaxima within the same cluster as the label. Analyses thresholded at Z > 2.3, cluster-corrected (p<0.05). | | | | | | | | | | | | | | | | | | | | | | | | |

| Table 12. MNI Coordinates positively correlated with Auditory SOR within ASD Group | | | | | |
| --- | --- | --- | --- | --- | --- |
|  | Post-Prime > Pre-Prime | | | | |
|  |  | MNI Peak (mm) | | | Max |
|  | Voxels | x | y | z | Z |
| **Right Putamen/Insula/Thalamus/Caudate** | 443 | 22 | 14 | 4 | 3.72 |
| **Bilateral Cerebellum (Lobule I-IV)** | 427 | 0 | -50 | -24 | 3.53 |
| **Left Hippocampus** | 293 | -12 | -40 | 4 | 3.26 |
| Note: x, y, and z refer to the left-right, anterior-posterior, and inferior-superior dimensions; Z refers to the Z-score at those coordinates; voxels refers to the number of voxels in the cluster. Labels without voxels indicate submaxima within the same cluster as the label. Analyses thresholded at Z > 2.3, cluster-corrected (p<0.05). | | | | | |


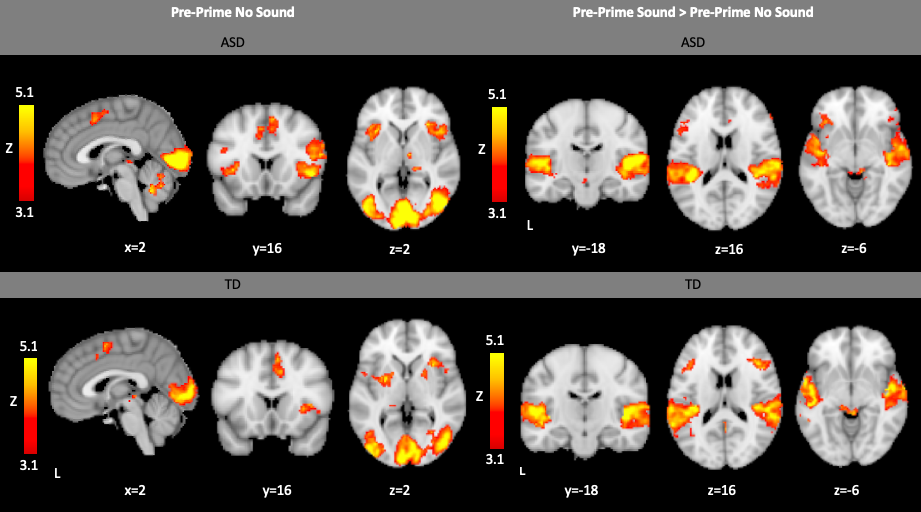


**Supplementary Figure 1:** Within-group results for the emotion identification task before participants saw the self-face prime (Pre-Prime No Sound) and changes with the addition of the auditory stimulus (Pre-Prime Sound > Pre-Prime No Sound). Contrasts thresholded at Z > 3.1, cluster-corrected (p<0.05).


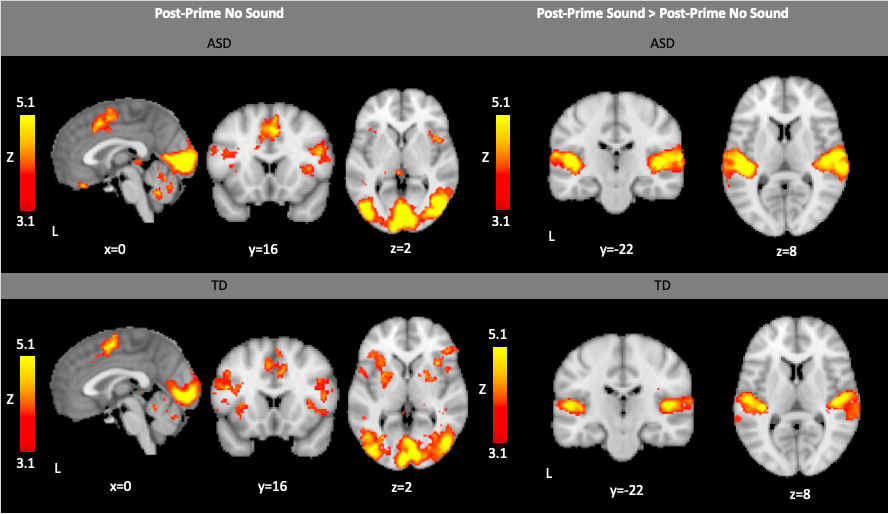


**Supplementary Figure 2:** Within-group results for the emotion identification task after participants saw the self-face prime (Post-Prime No Sound) and changes with the addition of the auditory stimulus (Post-Prime Sound > Post-Prime No Sound). Contrasts thresholded at Z > 3.1, cluster-corrected (p<0.05).


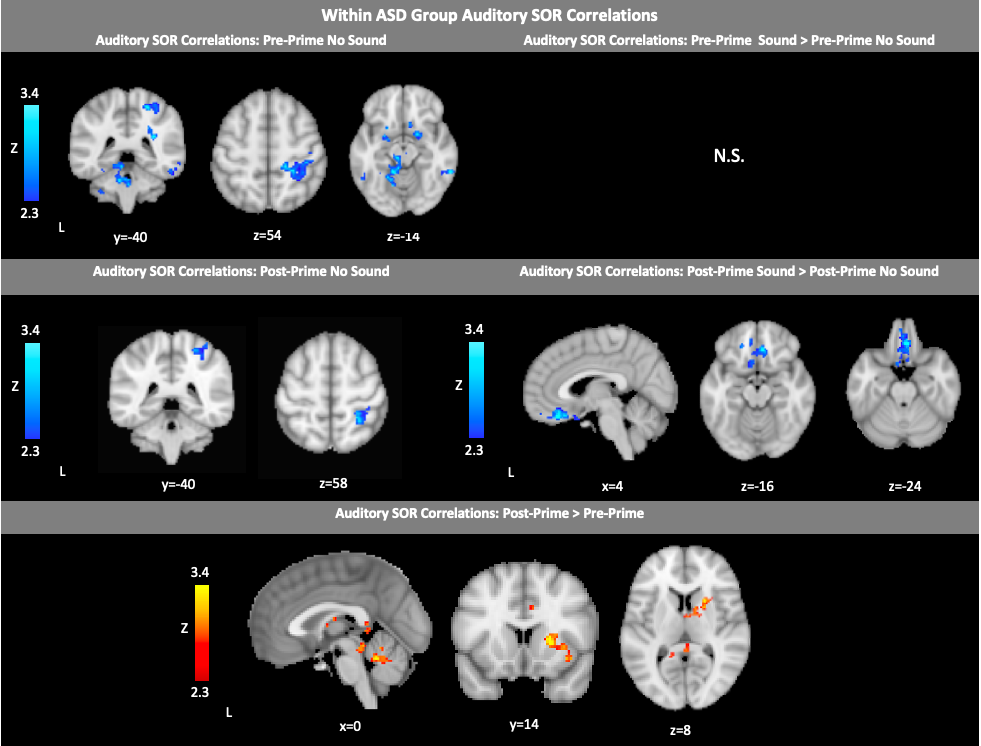


**Supplementary Figure 3:** Negative (blue) and positive (red) correlations with Auditory SOR during the emotion identification task within the ASD group. Contrasts thresholded at Z > 2.3, cluster-corrected (p<0.05).
